# Variceal Bleeding Prophylaxis: Variceal Banding or Propranolol

**DOI:** 10.1155/2000/75914

**Published:** 2000-08

**Authors:** Gregory Van Stiegmann

**Affiliations:** GI Tumor and Endocrine Surgery University of Colorado Health Sciences Centre 4200 East Ninth Avenue Denver Colorado 80262 USA


### Variceal Bleeding Prophylaxis: Variceal Banding or Propranolol

### ABSTRACT

*Sarin, S. K., Lamba, G. S., Kumar, M., Misra, A. and Murthy, N. S.*

***Background and Methods:*** **We compared propranolol therapy and endoscopic ligation for the primary prevention of bleeding from esophageal varices. This prospective, controlled trial included consecutive eligible patients who had large varices (> 5 mm in diameter) that were at high risk for bleeding. The patients were assigned to either propranolol therapy, at a dose sufficient to decrease the base-line heart rate by 25 percent, or variceal ligation, to be performed weekly until the varices were obliterated or so reduced in size that it was not possible to continue treatment.**
***Results:*** **Of the 89 patients, 82 of whom had cirrhosis of the liver, 44 received propranolol and 45 underwent variceal ligation. The mean (±SD) duration of follow-up in each group was 14 ± 9 and 13 ± 10 months, respectively. The mean time required to achieve an adequate reduction in the heart rate was 2.5 ± 1.7 days; the mean number of sessions needed to complete variceal ligation was 3.2 ± 1.1. After 18 months, the actuarial probability of bleeding was 43 percent in the propranolol group and 15 percent in the ligation group ($P = 0.04$). Twelve patients in the propranolol group and four in the ligation group had bleeding. Three of the four in the ligation group had bleeding before their varices had been obliterated. Nine patients in the ligation group had recurrent varices, a mean of 3.7 months after the initial treatment. Five patients in each group died; bleeding from the varices was the cause of death of four patients in the propranolol group and of three in the ligation group. There were no serious complications of variceal ligation; in the propranolol group, treatment was stopped in two patients because of side effects.**
***Conclusions:*** **In patients with high-risk esophageal varices, endoscopic ligation of the varices is safe and more effective than propranolol for the primary prevention of variceal bleeding. (N. Engl. J. Med., 1999, 340, 988–93.)**
*©1999, Massachusetts Medical Society.*

*Keywords:* Esophageal varices, variceal bleeding, variceal banding, variceal bleed prophylaxis

### PAPER DISCUSSION

Therapy for preventing a first variceal hemorrhage should be undertaken in the context of the natural history of patients with esophageal varices. Approximately 30 percent of patients with compensated cirrhosis (Child-Pugh class A and early class B) have esophageal varices as compared with approximately 60 percent of those with decompensated disease (class C and advanced class B) [1]. Overall, 25 to 30 percent of patients with known esophagal varices will bleed from them within a two year period [2]. A first variceal hemorrhage is associated with mortality as high as 30 to 50 percent and the risk of death is inversely related to liver function as measured by the Child-Pugh classification [1]. Patients with large varices, particularly those with red color signs, and decompensated cirrhosis have the greatest risk for a first hemorrhage and the greatest chance of death. Patients at high risk for variceal hemorrhage who are untreated may have as much as a 40 percent chance of experiencing hemorrhage by one year and 60 percent chance by two years [3].

There are currently two viable treatments for prevention of a first variceal hemorrhage. Drug therapy with propranolol and other non-specific beta adrenergic blocking agents reduces the hepatic venous pressure gradient (HVPG) (measured by catheterizing an hepatic vein and determining the difference between free hepatic vein pressure and wedged hepatic venous pressure) and has been shown to reduce the risk of a first variceal hemorrhage [4]. Reduction in risk for a first hemorrhage has not, however, been consistently associated with a reduction in mortality. Other drugs such as isosorbide-5-mononitrate may be of value alone or in combination with beta adrenergic blocking agents. The combination of nitrates with beta blockers reduces portal pressure more than either given alone; however, long-term effects of nitrate therapy are uncertain and may result in a higher incidence of liver failure, particularly in older patients [5]. A major drawback to drug therapy is the fact that patients must be compliant with a lifelong regimen and be able to tolerate the medication. Complications of drug therapy are usually not life threatening but approximately 20 percent of patients cannot tolerate (or have a contraindication to) propranolol. Moreover, of those treated, more than half may not achieve the desired 20 percent reduction in hepatic venous pressure gradient [6].

Endoscopic treatment as prophylaxis against a first variceal hemorrhage is not widely employed outside of Japan. Endoscopic sclerotherapy has been examined in multiple western studies which have produced heterogeneous results [7]. Most endoscopists in these countries have abandoned the prophylactic use of sclerotherapy because the technique has not proven consistently superior to no treatment at all. One large trial from the United States was halted early because patients in the prophylactic sclerotherapy arm experienced more complications and mortality than those in the sham endoscopy cohort [8].

Band ligation is now recognized as the treatment of choice for endoscopic prevention of recurrent hemorrhage from esophageal varices in patients who have previously bled from varices (secondary prophylaxis) [9]. Multiple prospective and randomized comparisons of endoscopic band ligation with sclerotherapy for secondary prophylaxis, as well as meta-analysis, confirmed that the newer treatment is associated with fewer complications, a lower incidence of recurrent hemorrhage and more rapid eradication of varices as well as a survival advantage [10]. The single disadvantage associated with endoscopic ligation therapy is a tendency for recurrence of esophageal varices once initial eradication of varices has been achieved.

Two large trials have compared endoscopic ligation with no treatment for primary prevention of variceal hemorrhage. Results from these trials showed that endoscopic ligation was superior to no treatment at preventing a first variceal hemorrhage [3, 11]. These studies were criticized because of failure to compare the endoscopic method with a cohort of patients treated with beta adrenergic receptor blockade.

Sarin *et al.*, report the first large study comparing endoscopic band ligation with beta adrenergic blockade for prevention of a first variceal hemorrhage [12]. Patients who had portal hypertension and large esophageal varices, predominately as a result of cirrhosis, were treated with either endoscopic ligation or propranolol. Patients in the two treatment arms were well matched and were followed for a mean of 13 months. Cirrhotics treated with endoscopic ligation experienced an actuarial probability of hemorrhage from varices of 15 percent as compared with 43 percent in the propranolol group ($p = 0.04$). Five patients in each cohort died. No serious complications occurred in either group.

The Sarin trial was criticized, in an editorial which accompanied its publication, because the cohort treated with propranolol had an inordinately high incidence of hemorrhage [13]. Burroughs suggested that the dose of the medication may have been too low (average 70 mg) as compared with the average dose used in other trials (123 mg) [14]. This is a plausible explanation for the poor showing in the propranolol group but it may not be the only one. We do not know, for example, the average height or weight of the patients in either treatment arm. This information is irrelevant for endoscopic treatment but if Sarin's patients were smaller and lighter than those in trials from other countries the 70 mg dose may have been perfectly appropriate (doses in the other trials ranged from 40–300 mg) [4].

Most previous trials of propranolol for prevention of a first variceal hemorrhage did not focus on high risk patients with large varices but included a more heterogeneous population only some of whom were at higher risk for a first bleeding. Only four of the nine widely cited trials specifically enrolled patients with medium or large sized varices [14]. More relevant perhaps, is the observation that only one of these nine beta blockade trials had more Child class C patients than the current study, two did not report Child classification, and four of the remaining six either had fewer than 10% Child class C patients or none at all [4]. The Sarin study contained approximately 50 percent Child B and 30 percent Child C patients in each arm. Fourteen of the 16 patients who experienced bleeding were those with advanced liver disease. It is widely agreed that patients with moderate and large size varices experience a lower incidence of first bleeding when treated with beta adrenergic blockade. The beneficial effect of drug therapy for those with advanced liver disease *and* large varices; however, may be less profound.

Contraindications and compliance are the two main shortcomings of pharmacological treatment. Sarin *et al.*, excluded eight otherwise eligible patients from his study because of contraindications to propranolol. Two additional patients were withdrawn during the study because of complications related to the medication. The current study determined compliance with the drug regimen by measuring heart rates and interviewing the patients at monthly intervals for three months and then at three month intervals. This methodology is consistent with other studies. It is reasonable to speculate, given the interval between compliance measurements and the fact that bleeding in the majority of propranolol treated patients occurred after more than four months of follow up, that some of the patients may not have taken the medication consistently or perhaps stopped taking it altogether, an act known to be associated with an increased risk of hemorrhage [15]. It is also reasonable to hypothesize (although my data is anecdotal) that ability to comply with a drug regimen is inversely proportional to the Child class of the patient. In contrast to the relatively late timing of hemorrhage in the propranolol patients, only one of the five patients in the endoscopic treatment arm of the Sarin study who bled, did so after the first two months of study. This finding confirms the commonly held view that endoscopic eradication of varices greatly diminishes the risk of hemorrhage.

What can we conclude from this and other trials that have examined endoscopic ligation as primary prophylaxis? The risk of a first variceal hemorrhage in patients treated with endoscopic ligation in four published trials comparing endoscopic ligation with other therapies was: 9%; 29%; 19% and 13% [3,11,16,17]. These results combined with those of the current study (15% incidence) are consistent with or superior to results obtained in propranolol treated patients in other randomized trials. Such cross-trial comparison is not rigorous enough to permit firm conclusions; however, the trend is clear. Endoscopic ligation has emerged as a viable alternative to beta blockade and other pharmacological treatments. Several additional large studies comparing the endoscopic method with drug therapies are needed before a final determination of the value of endoscopic ligation for primary prophylaxis can be made. It is also possible that the combination of pharmacological and endoscopic therapies may be superior to either treatment alone and studies aimed at answering this question should be encouraged.

Treatment of patients who are at high risk for a first variceal hemorrhage with endoscopic ligation now appears justified, especially when such patients have contraindications to or cannot tolerate or comply with a drug regimen. I believe that further study will reconfirm the endoscopic method is equal or superior to the pharmacological treatments available today. Physician or patient preference and cost will likely determine which treatment will dominate.

Gregory Van Stiegmann
Professor of Surgery
Head: GI Tumor and Endocrine Surgery
University of Colorado Health Sciences Centre
4200 East Ninth Avenue
Denver, Colorado 80262
USA
Fax: +303 315 5527

### References

D'Amico,, G. and Luca, A. (1997). Natural History: clinical-haemodynamic correlations: prediction of the risk of bleeding. *Baillieres Clin. Gastroenterol.*, **11**, 243–56.

Burroughs, A. K., D'Heygere, F. and McIntyre, N. (1986). Pitfalls in studies of prophylactic therapy for variceal bleeding in cirrhotics. *Hepatology*, **6**, 1407–13.

Lay, C.-S., Tsai, Y.-T., Teg, C.-Y., Shyu, W.-S., Guo, W.-S., Wu, K.-L. and Lo, K.-J. (1997). Is banding an acceptable treatment for varices that have not bled (prophylaxis)? *Hepatology*, **25**, 1346–50.

Shahi, H. M. and Sarin, S. K. (1998). Prevention of a first variceal bleed: an appraisal of current therapies. *Am. J. Gastroenterol.*, **12**, 2348–58.

Angelico, M., Carli, L., Piat, C., Gentile, S. and Capocaccia, L. (1997). Effects of isosorbide-5-mononitrate compared with propranolol on first bleeding and long-term survival in cirrhosis. *Gastroenterology*, **113**, 1632–39.

Garcia-Tsao, G., Groszman, R., Fisher, R. L., Conn, H. O., Atterbury, C. E. and Glickman, M. (1985). Portal pressure, presence of gastroesophageal varices and variceal bleeding. *Hepatology*, **5**, 419–24.

Pagliaro, L., D'Amico, G., Sorensen, T. I. *et al.* (1992). Prevention of first bleeding in cirrhosis: a meta-analysis of randomized trials of nonsurgical treatment. *Ann. Int. Med.*, **117**, 59–70.

The Veterans Affairs Cooperative Variceal Sclerotherapy Group. (1991). Prophylactic sclerotherapy for esophageal varices in alcoholic liver disease: a randomized, single blind, multicenter clinical trial. *N. Engl. J. Med.*, **324**, 1779–84.

Tait, I. S., Krige, J. and Terblanche, J. (1999). Endoscopic band ligation of esophageal varices. *Br. J. Surg.*, **86**, 437–46.

Laine, L. and Cook, D. (1995). Endoscopic ligation compared with sclerotherapy for treatment of esophageal variceal bleeding: a Meta-analysis. *Ann. Int. Med.*, **123**, 280–7.

Sarin, S. K., Guptan, R. C., Jain, A. K. and Sundaram, K. R. (1996). A randomized controlled trial of endoscopic variceal band ligation for primary prophylaxis of variceal bleeding. *Eur. J. Gastroenterol. Hepatol.*, **8**, 337–42.

Sarin, S. K., Lamba, G. S., Kumar, M., Misra, A. and Murthy, N. S. (1999). Comparison of endoscopic ligation and propranolol for prevention of variceal bleeding. *New. Engl. J. Med.*, **340**, 988–93.

Burroughs, A. K. and Patch, D. (1999). Primary prevention of bleeding from esophageal varices. *New. Eng. J. Med.*, **340**, 1033–5.

Poynard, T., Cales, P., Pasta, L. *et al.* (1991). Beta-adrenergic-antagonist drugs in the prevention of gastrointestinal bleeding in patients with cirrhosis and esophageal varices – an analysis of data and prognostic factors in 589 patients from four randomized clinical trials. *N. Engl. J. Med.*, **324**, 1532–8.

Lebrec, D., Bernuau, J., Rueff, B. and Benhamou, J. P. (1982). Gastrointestinal bleeding after abrupt cessation of propranolol administration in cirrhosis. *N. Engl. J. Med.*, **307**, 560.

Svoboda, P., Kantorova, I., Ochmann, J., Kozumplik, L. and Marsova, J. (1999). A prospective randomized controlled trial of sclerotherapy *vs.* ligation in the prophylactic treatment of high risk esophageal varices. *Surg. Endosc.*, **13**, 580–4.

De, B. K., Ghoshal, U., Das, T., Santra, A. and Biswas, P. K. (1999). Endoscopic variceal ligation for primary prophylaxis of oesophageal variceal bleed: preliminary report of a randomized controlled trial. *J. Gastroenterol. Hepatol.*, **14**, 220–4.

### When are Gallbladder Polyps Malignant?

### ABSTRACT

*Furukawa, H., Kosuge, T., Shimada, K., Yamamoto, J., Kanai, Y., Mukai, K., Iwata, R. and Ushio, K., Small Polypoid Lesions of the Gallbladder. Differential Diagnosis and Surgical Indications by Helical Computed Tomography. Arch Surg.*, **133**, *735–739.*

***Objectives:*** **To demonstrate the helical computed tomographic (CT) features of small polypoid lesions of the gallbladder and to establish a clinical strategy based on CT findings for the treatment of such lesions.**

**Design Validation cohort study.**

**Setting Tertiary care public hospital.**

**Patients Thirty-one patients with polypoid lesions of the gallbladder (≤3cm) underwent CT followed by resection.**

***Main Outcome Measure:*** **The detectability of the lesions on both unenhanced and enhanced CT and the configuration of the lesions on enhanced CT were prospectively evaluated in comparison with the histopathological findings.**

***Results:*** **Unenhanced CT detected 14 (45%) of the 31 lesions, whereas enhanced CT detected all of the lesions. The detection rates of the neoplastic**

